# PANoptosis in Bacterial Infections: A Double-Edged Sword Balancing Host Immunity and Pathogenesis

**DOI:** 10.3390/pathogens14010043

**Published:** 2025-01-08

**Authors:** Xiaoe He, Xiangyan Jiang, Jiayin Guo, Hui Sun, Jing Yang

**Affiliations:** Cuiying Biomedical Research Center, The Second Hospital & Clinical Medical School, Lanzhou University, Lanzhou 730030, China; hexiaoe1215@163.com (X.H.); jiangxy19@lzu.edu.cn (X.J.); 220220906411@lzu.edu.cn (J.G.); sunhui@lzu.edu.cn (H.S.)

**Keywords:** PANoptosis, programmed cell death, bacterial infection, bacterial infectious disease, host–pathogen interactions

## Abstract

PANoptosis is a newly identified programmed cell death pathway that integrates characteristics of apoptosis, pyroptosis, and necroptosis. It plays a dual role in the host immune response to bacterial infections. On one hand, PANoptosis acts as a protective mechanism by inducing the death of infected cells to eliminate pathogens and releasing pro-inflammatory cytokines to amplify the immune response. On the other hand, bacteria can exploit PANoptosis to evade host immune defenses. This dual nature underscores the potential of PANoptosis as a target for developing novel therapies against bacterial infections. This review summarizes the molecular mechanisms of PANoptosis, along with the crosstalk and integration of different cell death pathways in response to various bacterial pathogens. We also discuss the dual roles of PANoptosis in bacterial infectious diseases, including sepsis, pulmonary infections, and intestinal infections. Elucidating the molecular mechanisms underlying PANoptosis and how bacteria manipulate this pathway offers critical insights into host–pathogen interactions. These insights provide a foundation for designing targeted antibacterial strategies, modulating inflammation, and advancing precision medicine to improve clinical outcomes.

## 1. Introduction

Programmed cell death (PCD) is a fundamental mechanism that enables the innate immune system to combat microbial infections and maintain immune homeostasis [1]. The primary forms of PCD include apoptosis, pyroptosis, and necroptosis, each representing distinct cellular pathways that play critical roles in regulating immune responses. Apoptosis primarily induces non-inflammatory cell death through the activation of cysteinyl aspartate-specific proteinases (caspases/CASPs), leading to cell shrinkage and DNA fragmentation without triggering significant inflammation [2,3]. In contrast, pyroptosis and necroptosis are lytic forms of PCD that promote inflammation, aiding in pathogen clearance [4]. Pyroptosis is driven by gasdermin proteins and inflammatory CASPs, resulting in cell lysis and the release of pro-inflammatory cytokines, such as interleukin-1β (IL-1β) and IL-18 [4]. Necroptosis, mediated by receptor-interacting protein kinases 1 (RIPK1) and 3 (RIPK3) along with the pseudokinase mixed lineage kinase domain-like protein (MLKL), results in plasma membrane rupture and inflammatory responses [4].

PANoptosis, a recently identified form of inflammatory cell death, integrates features of pyroptosis, apoptosis, and necroptosis through the assembly of a molecular platform known as the PANoptosome [5]. This complex orchestrates the interplay among these pathways, forming a robust, multi-faceted defense system against pathogens [6]. Unlike single-pathway PCD, inhibition of apoptosis, pyroptosis, or necroptosis alone does not completely abrogate PANoptosis, highlighting its distinct and interconnected regulatory mechanisms [7,8]. Key components of the PANoptosome include Z-DNA binding protein 1 (ZBP1), RIPK1, RIPK3, CASP8, and the adaptor protein apoptosis-associated speck-like protein containing a CASP recruitment domain (ASC) [9]. These molecules are recruited in response to pathogen-associated molecular patterns (PAMPs), damage-associated molecular patterns (DAMPs), and cytokines, enabling the integration of cell death signals and fostering a coordinated immune response [10].

The interaction between host immune responses and bacterial pathogenesis is tightly regulated, with PANoptosis serving as a critical interface [11]. Bacterial pathogens such as *Salmonella* Typhimurium, *Listeria monocytogenes*, and *Yersinia* species can induce PANoptosis in host immune cells, influencing both pathogen clearance and immune modulation [11]. While the PANoptosome reinforces host defense by eliminating infected or compromised cells, bacteria can hijack this process to disrupt immune responses and promote their survival [11,12]. This dual nature of PANoptosis, both protective and detrimental, underscores its pivotal role in bacterial pathogenesis.

A comprehensive understanding of PANoptosis in bacterial infections is vital for developing novel therapeutic strategies. Targeting PANoptosis offers dual benefits: enhancing pathogen clearance through improved host defenses while minimizing tissue damage caused by excessive inflammation. Identifying specific molecular regulators of PANoptosis could facilitate the design of therapeutic agents to bolster immune responses, particularly against drug-resistant pathogens. Additionally, PANoptosis-related biomarkers hold promise for assessing patient prognosis and guiding personalized treatment approaches.

## 2. Molecular Mechanisms of PANoptosis

**Figure 1:**
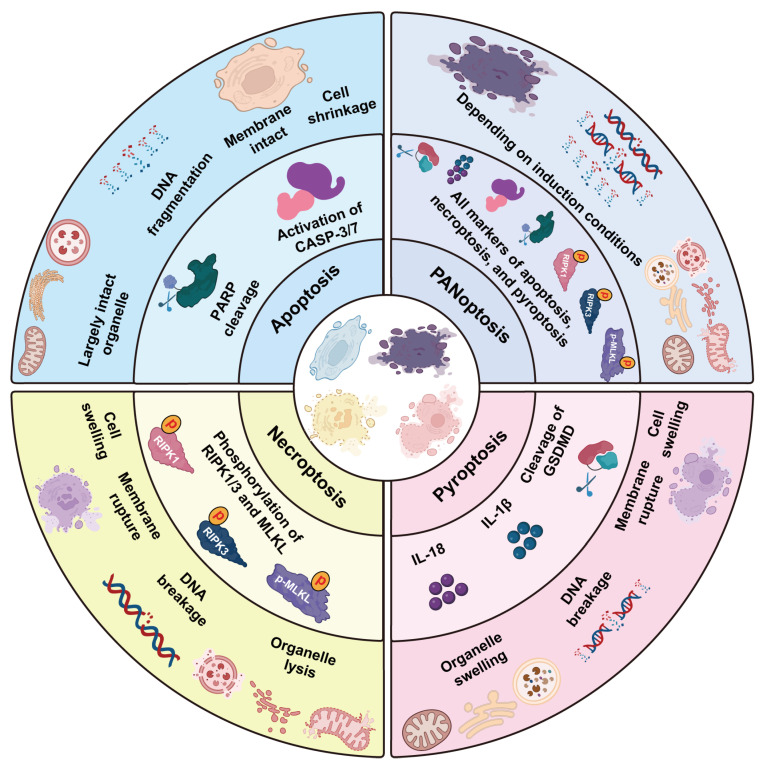
Cellular and biochemical features of pyroptosis, apoptosis, necroptosis, and PANoptosis. This diagram summarizes the key morphological, organelle, DNA, and biochemical changes in pyroptosis, apoptosis, necroptosis, and PANoptosis. Each cell death pathway is characterized by distinct alterations in cell shape, organelle integrity, DNA fragmentation, and the activation of specific biochemical markers. PANoptosis integrates features from all three pathways, exhibiting a variable combination of these changes depending on the specific induction conditions and the interplay of the involved molecular signals.

In 2016, Kanneganti and colleagues observed that macrophages infected with influenza A virus (IAV) undergo pyroptosis, apoptosis, and necroptosis simultaneously [13]. This discovery laid the foundation for the concept of “PANoptosis”, introduced in 2019 to describe a unique form of PCD that integrates elements of all three pathways and cannot be fully explained by any single mechanism [10] (Figure 1, Table 1). Subsequent studies identified the PANoptosome, a multi-component protein complex that acts as the central platform for coordinating the activation and regulation of these distinct cell death pathways [9]. Since its identification, PANoptosis has attracted extensive attention for its role in cancer, inflammation, and microbial infections [14], highlighting its importance in balancing effective immune responses and preventing excessive tissue damage.

### 2.1. Composition and Function of the PANoptosome Complex

The PANoptosome is analogous to inflammasomes, comprising three key components: sensor proteins, adaptor proteins, and effector proteins [15] (Table 2). Sensor proteins include NOD-like receptor pyrin domain-containing proteins (NLRPs) and NOD-like receptor caspase recruitment domain-containing proteins (NLRCs), absent in melanoma 2 (AIM2), ZBP1, etc. [15,16]. Adaptor proteins, such as ASC and Fas-associated death domain protein (FADD), provide the scaffold for complex assembly. Effector proteins, including various CASPs (e.g., CASP8, CASP1, CASP3), gasdermin D (GSDMD), RIPK1, RIPK3, and MLKL, execute cell death and inflammatory responses [15].

**Figure 2:**
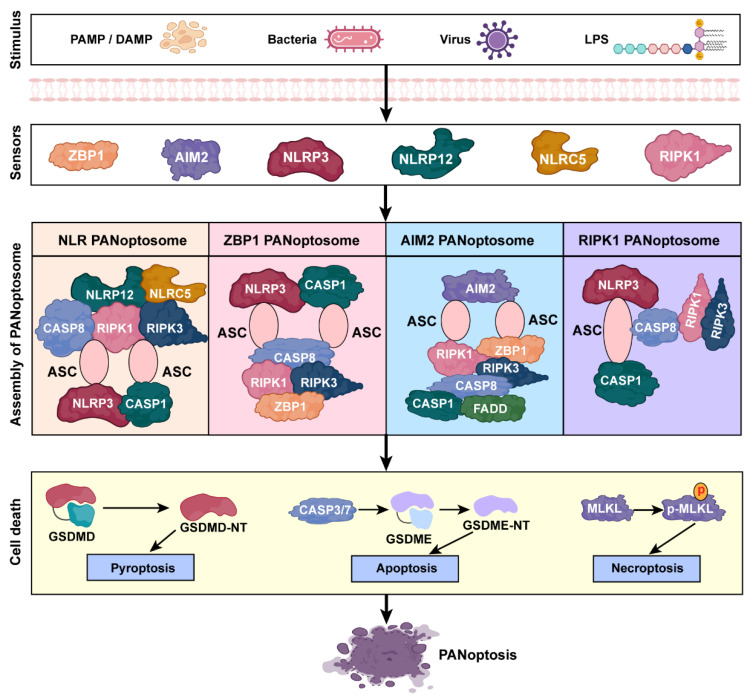
An overview of PANoptosis molecular mechanisms. The diagram illustrates the progression of PANoptosis, starting with various stimuli, including PAMPs, DAMPs, bacteria, viruses, and LPS. These triggers are recognized by specific sensors, such as NLRP3, ZBP1, AIM2, NLRP12, NLRC5, and RIPK1, leading to the assembly of distinct PANoptosomes, including the NLRP12-PANoptosome, ZBP1-PANoptosome, AIM2-PANoptosome, and RIPK1-PANoptosome. Each PANoptosome complex integrates key components such as ASC, caspase-1, caspase-8, RIPK1, and RIPK3, facilitating the activation of pyroptosis, apoptosis, and necroptosis. The convergence of these pathways culminates in the unified cell death process known as PANoptosis, which serves as a central mediator of regulated inflammatory cell death.

Upon detecting PAMPs or DAMPs, sensor proteins recruit adaptor proteins to form the scaffold of the PANoptosome. Effector proteins are subsequently engaged, mediating caspase activation, inflammasome assembly, and MLKL phosphorylation [32] (Figure 2). For instance, RIPK1 and RIPK3 are critical for transducing necroptotic signals, facilitating the phosphorylation and activation of MLKL, which ultimately leads to plasma membrane rupture [33]. CASP8 bridges apoptotic and pyroptotic pathways by cleaving substrates that drive both forms of cell death, such as the Bcl-2 homology 3 (BH3)-interacting domain death agonist (Bid) and GSDMD [34]. The adaptor protein ASC facilitates inflammasome assembly, promoting CASP1 activation and the maturation of pro-inflammatory cytokines IL-1β and IL-18 [35]. Together, these coordinated signaling events ensure a flexible and integrated response to pathogens and cellular stress.

The composition of the PANoptosome allows it to integrate and coordinate signals from multiple PCD pathways, ensuring a robust and multi-faceted cellular death response [36]. This integration helps to eliminate infected or damaged cells, thereby preventing pathogen replication and spread. It also regulates the intensity and duration of inflammatory responses, maintaining tissue homeostasis and preventing pathogens from evading host immune defenses through diverse cell death mechanisms [15]. However, dysregulation in the assembly or function of the PANoptosome can contribute to the onset and progression of various diseases [37]. Impaired PANoptosome function may lead to insufficient pathogen clearance, resulting in infectious diseases [38]. Conversely, its excessive activation can cause chronic inflammation and tissue damage [16]. Furthermore, abnormalities in cell death signaling may promote autoimmune responses or allow cancer cells to evade apoptosis, leading to autoimmune diseases or cancer [15].

### 2.2. Crosstalk and Integration Among Apoptosis, Pyroptosis, and Necroptosis

PANoptosis epitomizes the convergence of apoptosis, pyroptosis, and necroptosis, highlighting the complex interactions and regulatory interplay among these distinct PCD pathways. For example, during *Salmonella* infection, CASP8 inactivation by the effector protein *Salmonella* outer protein F (SopF) results in the inhibition of pyroptosis and apoptosis while promoting necroptosis, reflecting the interconnected regulation and adaptability among these pathways [39]. This interplay prevents pathogens from evading host defenses by targeting a single cell death pathway. The PANoptosome serves as a unified platform for these interactions, enabling a coordinated and effective cell death response [8].

Feedback mechanisms within PANoptosis further enhance this integration, ensuring that the activation of one pathway influences the others. This creates a balanced and adaptable cell death response, dynamically tailored to the pathogen type and the specific infection context [6,14].

### 2.3. Key Regulatory Molecules and Signaling Pathways

PANoptosis is regulated by a complex network of molecules and signaling pathways, including the CASP family, RIPK family, and inflammasome components [36].

CASP Family: CASP8 not only initiates apoptotic signaling but also mediates pyroptotic processes by cleaving GSDMD, a key executor of pyroptosis [40]. Additionally, CASP1 and CASP11 are crucial for triggering pyroptosis, leading to plasma membrane rupture and the release of pro-inflammatory cytokines such as IL-1β and IL-18 [41].

RIPK Family: RIPK1 and RIPK3 are critical mediators of necroptosis. Their interaction leads to the phosphorylation of MLKL, which oligomerizes and translocates to the plasma membrane, causing membrane disruption and inflammatory responses [42]. Within the context of PANoptosis, RIPK1 and RIPK3 also participate in the modulation of apoptosis and pyroptosis, thereby integrating necroptotic signals with other PCD pathways [14].

Inflammasome components, such as ASC, are essential for the formation of the PANoptosome. ASC facilitates the recruitment and activation of CASP1, which is crucial for pyroptosis and the maturation of pro-inflammatory cytokines [43]. The interplay between inflammasome activation and other PCD pathways, such as apoptosis and necroptosis, underscores the inflammatory nature of PANoptosis [44].

Furthermore, post-translational modifications, including ubiquitination and phosphorylation, are crucial for regulating the interactions and activities of PANoptosome components. For example, the ubiquitination of RIPK1 by E3 ligases, such as cellular inhibitor of apoptosis proteins 1 and 2 (cIAP1/2), inhibits necroptotic signaling. Conversely, RIPK1 deubiquitination by enzymes like CYLD lysine 63 deubiquitinase (CYLD) promotes PANoptosome assembly [2]. These modifications ensure precise control over the initiation and execution of PANoptosis, preventing excessive or insufficient cell death responses.

Cytokines, such as interferons (IFNs), can upregulate the expression of PANoptosome components, enhancing the cell’s capacity to respond to infections [20]. This cytokine-mediated regulation ensures the precise activation of PANoptosis in response to immune challenges, promoting effective pathogen clearance while minimizing the risk of cytokine-driven hyperinflammation, such as cytokine release syndrome (CRS), which can result from uncontrolled PANoptosis activation.

## 3. Mechanisms by Which Bacteria Induce PANoptosis

**Figure 3:**
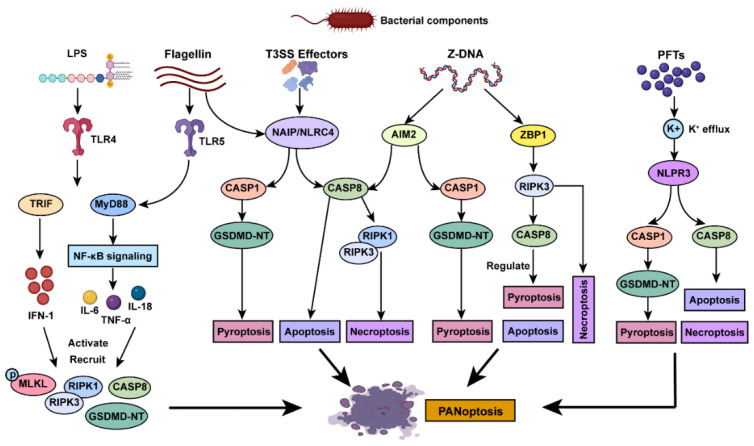
Initiation of PANoptosis during bacterial pathogen invasion. This schematic highlights the molecular mechanisms by which bacterial components trigger PANoptosis. Pathogen-associated molecules, such as LPS, flagellin, T3SS effectors, Z-DNA, and PFTs, activate host sensors including TLR4, TLR5, NAIP/NLRC4, AIM2, ZBP1, and NLRP3. These sensor proteins recruit key downstream adaptors and effectors such as caspase-1, caspase-8, RIPK1, and RIPK3, facilitating the induction of pyroptosis, apoptosis, and necroptosis. The convergence of these distinct cell death pathways results in the execution of PANoptosis, an integrated inflammatory cell death program essential for the host response to bacterial infections.

PANoptosis is crucial for eliminating infected cells and controlling the spread of the pathogen [45]. Bacteria induce PANoptosis through complex mechanisms involving the recognition of bacterial components by host PRRs, the action of bacterial toxins and effector proteins, and the manipulation of host cell signaling pathways [11] (Figure 3).

### 3.1. Activation of PRRs

Bacterial components are detected by host PRRs, including NOD-like receptors (NLRs), Toll-like receptors (TLRs), and cytosolic DNA sensors, which in turn initiate signaling pathways leading to PANoptosis [46]. (1) TLRs: Bacterial components such as lipopolysaccharide (LPS) and flagellin activate TLR4 and TLR5, respectively. These activations trigger downstream signaling cascades involving nuclear factor kappa B (NF-κB) and interferon regulatory factors (IRFs), promoting the expression of pro-inflammatory cytokines and interferons that prime PANoptotic pathways [47,48]. (2) NLRs: Intracellular bacterial components, including flagellin and toxins, activate NLRs such as NLRP3 and NLRC4. For example, *Salmonella* flagellin can activate both the NLRP3 and NAIP/NLRC4 inflammasomes, leading to the activation of CASP1 and subsequent pyroptosis [49]. (3) Cytosolic DNA sensors: Bacterial DNA in the cytosol can be detected by sensors such as AIM2 and ZBP1. During *Francisella novicida* infection, the DNA sensor cyclic GMP-AMP synthase (cGAS) and its adaptor protein stimulator of interferon genes (STING) induce the production of type I IFNs, which promote the expression of cell-intrinsic immunity effector proteins. This process results in bacteriolysis and the release of bacterial DNA, enabling its recognition by AIM2 [50].

### 3.2. Role of Bacterial Toxins and Effector Proteins

Bacteria produce various toxins and effector proteins that manipulate host cell death pathways to induce PANoptosis. (1) Pore-forming toxins (PFTs): PFTs, the largest group of bacterial toxins, are critical virulence factors [51]. For example, alpha-hemolysin (Hla) from *Staphylococcus aureus* and uropathogenic *Escherichia coli* creates pores in host cell membranes, leading to ion fluxes that activate the NLRP3 inflammasome and subsequent PANoptosis [52,53]. (2) Type III secretion system (T3SS) effectors: Gram-negative bacteria, such as *Yersinia enterocolitica*, *Salmonella enterica*, and *Shigella flexneri*, use T3SS to inject effector proteins into host cells upon sensing appropriate environmental signals. These effectors can modulate host signaling pathways to activate PANoptosis. For instance, *Shigella* employs effector proteins such as outer *Shigella* protein C1 (OspC1), OspC3, OspD3, and invasion plasmid antigen H7.8 (IpaH7.8) to block CASP8, CASP4/11, necroptosis, and GSDMD, respectively [54]. This manipulation disrupts individual PCD pathways while triggering the compensatory activation of alternative mechanisms, ultimately facilitating PANoptosis as a coordinated immune response to *Shigella* infection.

### 3.3. Specific Mechanisms Employed by Different Bacteria

Host cells recognize various bacterial components, triggering PANoptosis as a defense against bacterial infections. However, bacteria have evolved distinct mechanisms to either exploit or evade the immune responses associated with PANoptosis, thereby promoting their survival and dissemination.

(1) *Salmonella*: this pathogen utilizes its T3SS to activate PANoptosis primarily through the NLRC4 and NLRP3 inflammasomes. *Salmonella* flagellin and T3SS components such as PrgJ are recognized by NLRC4, resulting in CASP1 activation and pyroptosis [55]. Mitochondrial damage and potassium efflux activate the NLRP3 inflammasome, further amplifying pyroptosis [56]. *S.* Typhimurium activates death receptor (DR) signaling and PRRs, leading to CASP8 activation and the initiation of apoptosis. Additionally, CASP8 interacts with RIPK1 and RIPK3, which phosphorylate MLKL to induce necroptosis, ultimately converging on PANoptosis [57]. (2) *L. monocytogenes*: it primarily induces PANoptosis through the production of listeriolysin O (LLO), a pore-forming toxin. LLO disrupts phagosomal membranes, allowing the bacteria to escape into the cytosol [58]. Cytosolic bacterial DNA activates the AIM2 inflammasome, while LLO induces mitochondrial damage, leading to the activation of the NLRP3 inflammasome [58]. Both inflammasomes promote CASP1 activation and pyroptosis. During infection, pro-inflammatory cytokines such as tumor necrosis factor alpha (TNF-α) activate RIPK1, which interacts with RIPK3 to phosphorylate MLKL, causing membrane rupture and necroptosis, thereby contributing to the overall PANoptotic response. DR activation also triggers apoptosis, probably mediated by TNF-α and/or B-cell lymphoma-2 (Bcl-2), with these pathways collectively contributing to the induction of PANoptosis [59]. (3) *Yersinia* spp.: *Yersinia* employs the T3SS effector *Yersinia* outer protein J (YopJ) to inhibit the NF-κB and mitogen-activated protein kinase (MAPK) pathways by blocking transforming growth factor beta-activated kinase 1 (TAK1) activation, resulting in CASP8 activation and the induction of apoptosis [60]. Inhibition of TAK1 also sensitizes cells to necroptosis through the activation of RIPK3 and MLKL, consequently leading to PANoptosis. Importantly, *Yersinia*-induced cell death involves CASP8-mediated cleavage of GSDMD, a hallmark of pyroptosis, contributing to the inflammatory nature of this cell death response. Additionally, PRRs like TLRs recognize *Yersinia* components and activate inflammasomes like NLRP3, further enhancing pyroptosis through CASP1 activation and promoting the overall PANoptotic response [60]. (4) *S. aureus*: various PFTs, such as Hla and leukocidins (Luk), including Panton-Valentine leukocidin (PVL) and LukAB, contribute to PANoptosis induced by this bacterium, targeting epithelial and immune cells, respectively [61]. These toxins bind to host cell membranes and create pores in the plasma membrane, resulting in potassium efflux, thereby activating the NLRP3 inflammasome and CASP1, which initiate pyroptosis. Cell damage induced by these factors can trigger DR signaling, leading to CASP8 activation and apoptosis [61]. They also induce mitochondrial damage, leading to the release of cytochrome c and triggering apoptosis through the activation of CASP9 and CASP3 [62,63]. Additionally, pore formation induced by them can lead to the activation of RIPK1 and RIPK3, resulting in necroptosis [64]. The coordinated activation of pyroptosis, apoptosis, and necroptosis by these toxins constitutes PANoptosis, contributing to both pathogen clearance and tissue damage during infection. (5) *S. flexneri*: T3SS plays a crucial role in *S. flexneri*-induced PANoptosis. T3SS factors, such as MxiI and MxiH, activate the NAIP/NLRC4 inflammasome, triggering pyroptosis [65]. Notably, *Shigella* induces apoptosis by directly activating CASP1 via IpaB, bypassing the typical upstream signaling events and caspases that regulate CASP1 activation [66]. Additionally, common apoptosis ligands, such as TNF-α and factor-related apoptosis ligand (FasL), mediate necroptosis [67].

While inducing PANoptosis in host cells, these pathogens can also inhibit the crosstalk between multiple cell death pathways. *Shigella* serves as a prominent example of how pathogens target the crosstalk between apoptosis, necroptosis, and pyroptosis to disrupt coordinated host defenses. Studies indicate that apoptosis and necroptosis dynamically interact to orchestrate immune responses during late-stage infections. *Shigella* employs a dual inhibitory strategy by targeting key regulators, such as CASP8 and RIPK1/3, to uncouple these pathways, thereby facilitating immune evasion and promoting bacterial survival. Specifically, *Shigella* effector proteins, including OspC1 and OspD3, suppress apoptosis and necroptosis, respectively. OspC1 blocks CASP8, which not only prevents apoptosis but also relieves the inhibition of RIPK1/RIPK3 signaling, enabling necroptosis through MLKL activation. In contrast, OspD3 targets RIPK1 and RIPK3 for degradation, effectively blocking necroptosis and disrupting its crosstalk with apoptosis [68]. Simultaneously, *Shigella* inhibits pyroptosis through OspC3-mediated suppression of CASP4 activation [69]. Additional effectors, such as the virulence factor VirA and invasion plasmid gene D (IpgD), further suppress apoptosis via distinct mechanisms, including calpastatin degradation and phosphoinositide 3-kinase (PI3K)/protein kinase B (AKT) pathway activation, while LPS prevents CASP3/7 activation, ensuring apoptotic inhibition [70,71,72]. Collectively, these strategies highlight *Shigella*’s ability to dismantle the integrated regulatory networks governing programmed cell death, thereby suppressing crosstalk between pathways to maintain its replicative niche and evade immune clearance.

## 4. PANoptosis in Bacterial Infectious Diseases

PANoptosis integrates pyroptosis, apoptosis, and necroptosis, forming a coordinated cell death mechanism that is crucial for host defense against bacterial infections but can also contribute to disease pathology. Recent studies have highlighted the role of PANoptosis in various bacterial infections, such as bacterial sepsis, pulmonary bacterial infections, and intestinal infections, providing insights into its dual functions in immunity and disease progression (Table 3).

### 4.1. PANoptosis in Bacterial Sepsis

Bacterial sepsis is a life-threatening condition arising from an uncontrolled host response to infection, which can result in widespread inflammation and organ failure [79]. During sepsis, PAMPs like LPS from Gram-negative bacteria are detected by PRRs like TLR4, triggering downstream signaling pathways [79]. The formation of the PANoptosome complex during sepsis facilitates the coordinated activation of pyroptosis, apoptosis, and necroptosis [80]. Key molecules involved in these pathways include CASP1 (pyroptosis), CASP8 (apoptosis), and RIPK1 and RIPK3 (necroptosis) [14]. The activation of these pathways results in the secretion of pro-inflammatory cytokines such as IL-1β and IL-18, amplifying the inflammatory response [14]. PANoptosis eliminates invading bacteria by triggering the death of infected cells through multiple pathways, thus reducing tissue damage caused by bacterial replication [81]. However, excessive activation of PANoptosis can result in immunopathology, including lymphopenia, multi-organ failure, and septic shock, which aggravate the severity of sepsis and increase the risk of secondary infections [82,83,84].

Researchers have identified 16 PANoptosis-related genes associated with sepsis using single-cell RNA sequencing and chipset data, which were used for consensus clustering and functional enrichment analysis [73]. The study classified sepsis cases into three distinct PANoptosis clusters, revealing variations in immune infiltration and survival outcomes among the subgroups. The findings also showed that the PANscore, derived from PANoptosis-related genes, could predict the prognosis of sepsis patients, highlighting the roles of specific genes like ZBP1, X-linked inhibitor of apoptosis (XIAP)-associated factor 1 (XAF1), interferon-induced protein 44-like (IFI44L), suppressor of cytokine signaling 1 (SOCS1), and poly(ADP-ribose) polymerase family member 14 (PARP14) in the immune response in sepsis. The study further emphasized the involvement of IFI44 and IFIH1 in regulating immune responses in sepsis, with radical S-adenosyl methionine domain containing 2 (RSAD2) specifically playing a role in PANoptosis within the septic environment. Furthermore, they indicated that the high PANscore group was associated with better survival outcomes, emphasizing the importance of PANoptosis regulation in determining the severity and prognosis of sepsis. This scoring system provides a potential prognostic tool for sepsis patients, aiding clinicians in risk stratification and treatment adjustment. By identifying specific clusters among sepsis patients, the study highlighted the variability in immune responses, which is crucial for personalized therapeutic approaches. The stratification of PANoptosis clusters revealed distinct immune infiltration profiles, which provide insights into the relationship between PANoptotic molecular mechanisms and patient outcomes. These findings are valuable for understanding the immune landscape of sepsis and developing targeted interventions. However, further experimental validation and clinical feasibility studies are needed to assess the translational potential. Expanding patient diversity and incorporating additional immune markers could further enhance the comprehensiveness and applicability of the findings.

Septic lung injury (SLI) is a major complication of sepsis, associated with high mortality rates and currently lacking effective pharmacological treatments. In SLI, activated NLRP3 or AIM2 inflammasomes can initiate apoptosis through CASP8 activation, subsequently activating effector CASPs like CASP3/7. Inhibition of CASP8 promotes RIPK3-mediated necroptosis through MLKL phosphorylation, further enhancing lung inflammation. This interconnected mechanism not only leads to extensive cell death but also aggravates lung damage by increasing vascular permeability, disrupting tissue integrity, and recruiting more inflammatory cells [85]. Furthermore, a study combining bioinformatics analysis with experimental validation demonstrated that four signature genes—cluster of differentiation (CD14), GSDMD, IL-1β, and FAS—are highly involved in immune responses and multiple inflammatory pathways in SLI [74]. Of these, CD14, FAS, and IL-1β were suggested to participate in PANoptosis, driving the progression of acute lung injury (ALI). Additionally, research has shown that miR-29a-3p suppresses PANoptosis and inflammatory responses in alveolar epithelial cells by targeting tumor necrosis factor receptor 1 (TNFR1), thereby alleviating lung injury in ALI mice. This indicates that miR-29a-3p may serve as a potential therapeutic target for the treatment of ALI [75]. Moreover, inhibition of cIAP1/2 reduces RIPK1 phosphorylation in pulmonary endothelial cells and mitigates SLI [86]. Additional evidence suggests that Dachengqi decoction-dispensing granules may alleviate LPS-induced PANoptosis by inhibiting the ZBP1-RIPK1-PANoptosome pathway, significantly reducing excessive inflammation and epithelial cell damage during the SLI process, making it a promising candidate for treating SLI [87]. The crosstalk between these cell death pathways underscores the complexity of SLI, where blocking a single pathway is often insufficient for complete protection. Effective therapeutic strategies may need to target multiple PCD pathways simultaneously to mitigate the severity of SLI and improve outcomes in sepsis patients.

Sepsis-associated encephalopathy (SAE) is a serious complication of sepsis that leads to neuronal cell death, resulting in deterioration of mental status and cognitive function. In a neonatal rat model of SAE, researchers identified PANoptosis in cortical neurons, revealing that apoptosis and pyroptosis were predominantly present in the cortex, followed by a complementary and balanced necroptotic response [76]. All three subfamilies of the MAPK pathway were found to be activated in SAE, with the p38 MAPK signaling pathway regulating all three PANoptotic pathways. Blocking p38 MAPK activation by SB203580 promoted necroptosis while inhibiting both apoptosis and pyroptosis. Moreover, the MAPK regulatory factor TLR9 was found to modulate PANoptosis through the p38 MAPK pathway. Downregulation of TLR9 using ODN2088 enhanced survival rates and mitigated pathological alterations in SAE rats [76]. Previous studies have indicated that TLR9 has a detrimental effect on cardiac function during sepsis [88], and another study reported that TLR9/Caveolin-1 signaling may help predict treatment outcomes in sepsis patients [89]. Collectively, these findings suggest that TLR9 could be a potential therapeutic target for treating SAE, as well as a predictive biomarker for treatment outcomes in sepsis.

### 4.2. PANoptosis in Pulmonary Bacterial Infections

Pulmonary bacterial infections caused by pathogens such as *Streptococcus pneumoniae*, *S. aureus*, *Yersinia pestis*, *Salmonella* Typhi, and *L. monocytogenes* can lead to conditions like pneumonia, sepsis, and severe tissue damage [90,91,92]. In the early stages, these infections are often associated with acute respiratory distress syndrome (ARDS) and can trigger PANoptosis [15]. The role of PANoptosis in bacterial pneumonia is dual-faceted: while it aids in the clearance of infected alveolar macrophages and epithelial cells, thereby reducing bacterial load, the excessive cell death and pro-inflammatory cytokine release also contribute to lung damage, edema, and impaired gas exchange. The death of alveolar epithelial cells is a hallmark of acute pneumonia progression, and severe PANoptosis can lead to ALI and multi-organ failure, such as ARDS and systemic inflammatory response syndrome (SIRS) [93].

Dysregulated inflammatory cytokines are a major contributor to ALI, with IFN-γ playing a key role in PANoptosis, inflammation, and tissue homeostasis [93]. In pneumonias caused by *S. aureus*, *S. pneumoniae*, and *Actinobacillus pleuropneumoniae*, IFN-γ exhibits diverse functions, including driving cytokine storms characterized by excessive production of TNF-α, IL-1β, and IL-6, leading to fatal lung injury. Additionally, IFN-γ signaling also influences immune cell infiltration in the lung, particularly alveolar macrophages and αβ T cells, resulting in delayed bacterial clearance [94,95]. A study has shown that IFN-γ deficiency increases the survival rate of mice infected with *A. pleuropneumoniae* by promoting IL-18 secretion in lung tissue. This led to elevated polymorphonuclear neutrophil-I (PMN-I) chemotaxis and inhibited PMN-II polarization, thereby enhancing the bactericidal function of PMNs [77]. In a mouse model of acute pneumonia induced by *Pasteurella multocida* toxin (PMT), lung levels of IFN-γ, its receptor, and associated proteins were significantly elevated. Studies have shown that in a lethal pneumonia model induced by PMT, IFN-γ independently facilitates the induction of PANoptosis, which is a critical pathogenic mechanism in *P. multocida* pneumonia. In IFN-γ-deficient mice, the levels of PANoptosis regulators, such as GSDMD, phosphorylated MLKL (pMLKL), and cleaved CASP3, were significantly reduced, with concurrent mitigation of lung tissue injury and organ dysfunction, resulting in improved survival rates [93]. The IFN-γ-induced PANoptosis activation and the subsequent cytokine storm represent potential therapeutic targets for treating bacterial pneumonia.

Pyroptosis and necroptosis significantly contribute to lung damage during bacterial pneumonia [96]. A study highlights the crucial role of the NLRP6 inflammasome in responding to *S. aureus* infection, particularly in mediating PANoptosis activation and exacerbating lung inflammation and tissue damage [78]. Hla acts as a key pathogenic factor that activates the NLRP6 inflammasome in alveolar macrophages and epithelial cells, resulting in the activation of downstream CASP1 and triggering pyroptosis. Concurrently, NLRP6 induces necroptosis via RIPK3 and MLKL phosphorylation, triggering inflammation and tissue injury. These mechanisms collectively contribute to the activation of PANoptosis, promoting the release of pro-inflammatory cytokines and recruiting neutrophils for bacterial clearance. However, NLRP6 activation also reduces IFN-γ production by NK cells, which impairs neutrophil oxidative burst and lowers bacterial clearance efficiency. Since IFN-γ is crucial for enhancing neutrophil bactericidal activity, reduced IFN-γ levels due to NLRP6 activation negatively impact the immune response to bacterial infection. This dysregulation between pro-inflammatory and anti-inflammatory signals results in excessive tissue damage, including increased vascular permeability, alveolar edema, and impaired gas exchange—key features of ALI and ARDS. The findings suggest that PANoptosis plays a dual role in bacterial pneumonia: contributing both to bacterial clearance and to increased inflammation and lung damage. If the systemic inflammatory response is not properly regulated, it can lead to multi-organ dysfunction and higher morbidity and mortality. Thus, therapeutic strategies should aim to modulate PANoptosis to achieve a balance between effective pathogen clearance and minimizing lung damage, ultimately improving outcomes for patients with bacterial pneumonia.

### 4.3. PANoptosis in Intestinal Infections

The intestinal inflammatory response is a critical component of host–pathogen interactions. Intestinal epithelial cells (IECs) serve as a barrier that protects the mucosa from pathogen invasion while coordinating various innate immune responses, thereby playing a key role in limiting the dissemination of foodborne pathogens [97]. The expulsion of infected IECs is a common defense mechanism against intestinal pathogen infections, effectively restricting pathogen proliferation within the intestinal epithelium [54]. PANoptosis is a crucial mechanism in this host defense.

For instance, *S.* Typhimurium, upon infecting the human host, can induce intestinal inflammation. The occurrence of pyroptosis and apoptosis in IECs promotes the expulsion of infected cells, which helps to limit bacterial replication and maintain the integrity of the intestinal barrier [98,99]. However, necroptosis can lead to disruption of the intestinal barrier and facilitate the spread of bacteria to the lamina propria and even systemically [100]. The release of pro-inflammatory cytokines and DAMPs exacerbates mucosal inflammation, which can lead to pathological conditions such as colitis. However, certain pathogens have evolved countermeasures to modulate this inflammatory response and evade host defenses. Research indicates that *Salmonella* manipulates IEC PANoptosis through the T3SS effector protein SopF, allowing it to evade host epithelial defenses [39]. Specifically, SopF activates phosphoinositide-dependent protein kinase-1 (PDK1), leading to the phosphorylation of p90 ribosomal S6 kinase (RSK), which inhibits CASP8 activation. This inhibition, in turn, suppresses pyroptosis and apoptosis in IECs while promoting necroptosis. The suppression of pyroptosis and apoptosis by SopF hinders the expulsion of infected IECs, while its induction of necroptosis compromises the intestinal barrier and enhances the internalization of *Salmonella*, facilitating bacterial translocation and systemic dissemination [39]. Although this series of events may alleviate local intestinal inflammation, it exacerbates systemic infection. Moreover, SopB suppresses host cell PANoptosis by activating the AKT signaling pathway, which may contribute to reducing inflammation and alleviating the severity of colitis caused by *Salmonella* infection [101,102,103]. The mechanisms by which SopF and SopB influence the fate of IECs through the modulation of PANoptosis offer promising therapeutic strategies for controlling *Salmonella* infections. The roles of additional known or unknown T3SS effectors of various Gram-negative bacteria in manipulating PANoptosis present significant avenues for further research.

## 5. Conclusions and Prospects

In summary, PANoptosis has emerged as a crucial regulatory mechanism in bacterial infectious diseases, significantly influencing immune responses, pathogen clearance, and the pathological outcomes of infections. The intricate interplay among pyroptosis, apoptosis, and necroptosis underscores the complexity of PANoptosis and its therapeutic potential. This mechanism serves as both a host defense against bacterial infections and a process exploited by bacteria to evade host immunity and promote their survival.

The manipulation of PANoptosis by pathogenic bacteria has garnered increasing attention, as it plays a critical role in bacterial survival within the host environment. For example, bacterial virulence factors, such as pore-forming toxins from Gram-positive bacteria and T3SS effectors from Gram-negative bacteria, can alter the PANoptosis process to subvert host defense responses, suppress immune activation, and facilitate bacterial persistence and evasion.

Future research should aim to elucidate the precise molecular mechanisms by which bacterial pathogens induce or manipulate PANoptosis, with a particular focus on the interactions between bacterial virulence factors and host immune signaling pathways. A deeper understanding of these processes could uncover potential therapeutic targets within the PANoptosome complex, paving the way for the development of PANoptosis modulators. Selective targeting of PANoptosis activation and the release of associated cytokines could optimize therapeutic outcomes, balancing effective pathogen clearance with the prevention of excessive inflammation and tissue damage. This approach holds significant promise for advancing precision medicine in bacterial infections by tailoring treatments to the specific pathogens and host responses.

Moreover, developing compounds that can induce PANoptosis in infected cells may provide an alternative or complement to traditional antibiotics, reducing reliance on high-dose therapies and minimizing associated side effects. Furthermore, investigating PANoptosis-related biomarkers for diagnostic and prognostic purposes could further enhance clinical decision-making. By stratifying patients based on disease risk and their response to PANoptosis-targeted therapies, personalized treatment strategies could significantly improve patient outcomes.

To fully realize the therapeutic potential of PANoptosis, future efforts should integrate basic research with translational studies. A comprehensive understanding of PANoptosis regulation across diverse bacterial infections will be instrumental in developing innovative treatment paradigms. These advancements hold the promise of improving the management of bacterial infectious diseases and addressing the escalating challenge of antimicrobial resistance.

## Figures and Tables

**Table 1:** A comprehensive overview of the characteristics of pyroptosis, apoptosis, necroptosis, and PANoptosis.

Characteristic	Pyroptosis	Apoptosis	Necroptosis	PANoptosis
**Morphology**	Cell swelling.	Cell shrinkage.	Cell swelling	Depending on induction conditions.
**Membrane integrity**	Rupture, pore formation and cytoplasmic leakage.	Intact, without pore formation and cytoplasmic leakage.	Rupture, pore formation and cytoplasmic leakage.	Depending on induction conditions.
**Nucleus**	DNA breakage.	DNA fragmentation, forming apoptotic bodies.	DNA breakage.	Depending on induction conditions.
**Organelles**	Significant swelling, especially of mitochondria.	Intact, though the cytoplasm condenses as the cell shrinks.	Organelle lysis, notably of mitochondria.	Mixed PCD features in the organelle.
**Biochemical Markers**	Cleavage of GSDMD, maturation, and release of IL-1β and IL-18.	Activation of CASP3/7, PARP cleavage, DNA laddering.	Phosphorylation of RIPK1/3 and MLKL membrane aggregation.	All of the above three PCD pathway markers.
**Inflammatory Response**	Strong inflammation with release of multiple cytokines.	Non-inflammatory and immunologically silent.	Causes an inflammatory response, release of DAMPs.	Strong inflammation, integrating multiple cell death pathways.
**Physiological Role**	Defenses against intracellular pathogens by eliminating infected cells and triggering inflammation.	Maintains tissue homeostasis, removes damaged or unnecessary cells without inflammation.	Backup mechanism for cell death involved in inflammatory diseases when apoptosis is inhibited.	Host defense mechanism that eliminates infected cells through multiple cell death pathways.
**Pathological Implications**	Excessive pyroptosis can lead to inflammatory diseases such as sepsis and atherosclerosis.	Dysregulation can result in cancer, autoimmune diseases, and neurodegenerative disorders.	Abnormal necroptosis is linked to inflammation, neurodegeneration, ischemia, and cancer.	Dysregulated PANoptosis drives inflammation, cancer, and infection severity.

**Table 2:** Composition and function of the PANoptosome complex.

Category	Molecule	Function	References
**Sensors**	AIM2	Detects cytosolic DNA, forms inflammasome with ASC, activates CASPs for pyroptosis.	[17]
	NLRP3	Inflammasome component that can be part of the PANoptosome under certain stimulatory conditions, linking inflammation with cell death.	[13,18,19]
	NLRP12	Detects PAMPs and stress signals, promoting inflammasome assembly and PANoptosome activation to regulate inflammation and cell death.	[20]
	NLRC5	Senses infections and inflammatory signals, activating inflammasomes and integrating into PANoptosome complexes to control immune responses and cell death.	[16]
	ZBP1	Serves as a sensor for viral components and mediates the assembly of PANoptosome with RIPK1 and RIPK3, triggering PANoptosis.	[21]
**Adaptors**	ASC	Adaptor protein that assembles inflammasome and PANoptosome, bridging sensors to CASPs.	[22]
	FADD	Adaptor linking death receptors to CASPs, modulates apoptosis and necroptosis pathways.	[23]
**Effectors**	CASP1	Initiates pyroptosis, cleaves GSDMD, and releases inflammatory cytokines.	[24]
	CASP8	Dual role in apoptosis and necroptosis, can switch roles depending on cellular context and signals. Functions as a catalytic effector in the complex, critical for executing apoptosis and pyroptosis, often in conjunction with RIPK1 and FADD.	[2,25]
	MLKL	Executes necroptosis by translocating to and disrupting the plasma membrane.	[26]
	GSDMD	Executes pyroptosis by forming pores in the cell membrane after cleavage by inflammatory CASPs.	[27,28]
	RIPK1	Acts as a scaffold and regulatory molecule that modulates both necroptosis and apoptosis, and is essential for NLRP3 inflammasome activation in certain contexts.	[29,30]
	RIPK3	Partners with RIPK1 and ZBP1 in PANoptosome to promote cell death pathways, including necroptosis.	[31]

**Table 3:** PANoptosis in bacterial infectious diseases: mechanisms and consequences for disease outcomes.

Disease	Findings	Impact of PANoptosis on Disease Outcomes	References
Sepsis	Identified 16 genes defining sepsis subtypes; RSAD2 is key in PANoptosis.	PANscore, derived from these genes, can stratify sepsis patients and guide treatment.	[73]
SLI	Identified PANoptosis-related genes (CD14, GSDMD, IL-1β, and FAS) in SLI, with high expression and diagnostic potential.	CD14, FAS, and IL-1β are involved in PANoptosis, driving the progression of ALI.	[74]
ALI	MiR-29a-3p is reduced in ALI, and its upregulation suppresses PANoptosis and inflammation via TNFR1 targeting.	MiR-29a-3p suppresses PANoptosis, alleviating lung injury and highlighting its potential as a therapeutic target for ALI.	[75]
SAE	TLR9 regulates PANoptosis of SAE through p38 MAPK signaling.	TLR9 inhibition reduced PANoptosis, improved survival, and mitigated brain damage.	[76]
Pneumonia	IFN-γ promotes PANoptosis in pneumonia by activating CASP3, GSDMD, and MLKL.	IFN-γ promotes PANoptosis, worsening lung injury, while its absence improves survival.	[77]
Pneumonia	NLRP6 activation in *S. aureus* pneumonia drives pyroptosis and necroptosis, disrupting neutrophil function and amplifying inflammation.	PANoptosis driven by NLRP6 worsens lung injury and inflammation, leading to increased mortality and impaired immune defense.	[78]
Intestinal inflammation	SopF inhibits CASP8, promoting necroptosis and suppressing pyroptosis and apoptosis in IECs.	SopF disrupts cell death pathways, promoting bacterial spread and worsening systemic infection.	[39]

## Data Availability

Not applicable.
